# The Plasma Membrane Ca^2+^-ATPase2 (PMCA2) Is Involved in the Regulation of Purkinje Cell Dendritic Growth in Cerebellar Organotypic Slice Cultures

**DOI:** 10.1155/2013/321685

**Published:** 2013-09-29

**Authors:** Pradeep Sherkhane, Josef P. Kapfhammer

**Affiliations:** Anatomical Institute, Department of Biomedicine, University of Basel, Pestalozzistraße 20, 4056 Basel, Switzerland

## Abstract

Purkinje cells are the principal neurons of the cerebellar cortex and have an extensive and elaborate dendritic tree. Chronic activation of type I metabotropic glutamate receptors inhibits Purkinje cell dendritic growth in organotypic cerebellar slice cultures. This effect is mediated by calcium influx through P/Q-type and T-type Ca^2+^ channels. We have now studied the role of the plasma membrane Ca^2+^-ATPase2 (PMCA2), a major calcium extrusion pump, for Purkinje cell dendritic development. We found that PMCA2 is strongly expressed in the plasma membrane and dendritic spines of Purkinje cells in organotypic slice cultures compatible with a role for controlling the local dendritic calcium equilibrium. Inhibition of PMCA2 activity by carboxyeosin resulted in a moderate reduction of Purkinje cell dendritic tree size indicating that the extrusion of calcium by PMCA2 is important for maintaining the dendritic calcium concentration and controlling dendritic growth. When inhibition of PMCA2 was combined with stimulation of type I metabotropic glutamate receptors, it partially rescued dendritic morphology. This protection can be explained by a compensatory inactivation of voltage-gated calcium channels in Purkinje cells after PMCA2 inhibition. Our results demonstrate that PMCA2 activity is an important regulator of the dendritic calcium equilibrium controlling Purkinje cell dendritic growth.

## 1. Introduction

Purkinje cells are the principal neurons of the cerebellar cortex and have an extensive and elaborate dendritic tree. They receive excitatory synaptic input from granule cell derived parallel fibers and inferior olive derived climbing fibers. The development of the Purkinje cell dendritic tree is controlled by a variety of intrinsic and extrinsic signals [1, 2]. We have previously shown that chronic activation of either type I metabotropic glutamate receptors (mGluR1s) or protein kinase C (PKC) in organotypic cerebellar slice cultures severely inhibits the growth and development of the Purkinje cell dendrites [3–5]. The stunted dendritic growth seen after mGluR1 or PKC stimulation is partially rescued by pharmacological blockade of P/Q-type and T-type Ca^2+^ channels, indicating that activation of these channels mediating Ca^2+^ influx contributes to the inhibition of Purkinje cell dendritic growth [6]. Besides the influx of calcium through voltage-dependent channels, calcium clearance mechanisms also affect the calcium equilibrium in Purkinje cells [7–9]. The plasma membrane Ca^2+^-ATPase2 (PMCA2) is reported to be involved in extrusion of calcium and cerebellar synapse function [7].

PMCA2 belongs to the family of P-type primary ion transport ATPases characterized by the formation of aspartyl phosphate intermediate during an ATP hydrolysis reaction cycle. Of the known PMCA variants, PMCA1 and PMCA4 are expressed ubiquitously whereas PMCA2 and PMCA3 are expressed prevalently in the central nervous systems. The PMCA2 isoform is highly expressed in the cerebellum, particularly in Purkinje cell dendrites and dendritic spines [10, 11]. Two spontaneous mouse mutants with a loss of function of PMCA2 [12, 13] and a PMCA2 knockout mouse [14] are known. They are characterized by a combination of deafness with a marked cerebellar ataxia. 

The aim of this study was to investigate whether PMCA2 activity may be involved in Purkinje cell dendritic growth and whether it would be modulating the effects of mGluR1 activation on the development of the Purkinje cell dendritic tree. PMCA2 can be pharmacologically inhibited by treatment with 5(6)-Carboxyeosin diacetate ester, shortly known as carboxyeosin. We have studied the effect of inhibiting PMCA2 by carboxyeosin on Purkinje cell dendritic growth and tested whether carboxyeosin treatment might modulate the reduction of the Purkinje cell dendritic tree seen after mGluR1 activation.

## 2. Materials and Methods

### 2.1. Organotypic Slice Cultures

Animal experiments were carried out in accordance with the European Communities Council Directive of 24 November 1986 (86/609/EEC) and were reviewed and permitted by Swiss authorities. Cultures were prepared from B6CF1 mice (CB6) as described previously [15–17]. Mouse pups were decapitated at postnatal day 8 and their brains were dissected aseptically. The cerebellum was separated in ice-cold preparation medium (minimal essential medium (MEM), 1% GlutaMAX (Gibco, Invitrogen), and pH 7.3) and slices of 350 *μ*m thickness were cut with a McIlwain tissue chopper under sterile conditions. Cerebellar slices were separated, transferred on to a permeable membrane (Millicell-CM, Millipore), and incubated with incubation medium (50% MEM, 25% Basal Medium Eagle, 25% horse serum, 1% GlutaMAX, and 0.65% glucose) with 5% CO_2_ at 37°C. The medium was refreshed every 2-3 days. The following pharmacological compounds were added to the medium at each change for a total of 7 days, starting at 2–4 days in vitro (DIV): (RS)-3,5-Dihydroxyphenylglycine (DHPG, Tocris, Bristol, United Kingdom) and 5(6)-Carboxyeosin diacetate ester (carboxyeosin, Molecular Probes, USA). The following concentrations were used: 10 *μ*M DHPG, 10 *μ*M and 20 *μ*M carboxyeosin. When carboxyeosin was used in combination with DHPG, it was added 24 h prior to the first DHPG treatment. Slices were kept in culture for a total of 9–11 days before fixation and immunohistochemical staining.

### 2.2. Immunohistochemistry

At DIV, 9–11 cultures were fixed in 4% paraformaldehyde for 6–24 hours at 4°C. All reagents were diluted in 100 mM phosphate buffer (PB), pH 7.3. Antibodies were added to the slices in fresh blocking solution (PB + 3% nonimmune goat serum + 0.5% Triton X-100) and incubated overnight at 4°C. After washing in PB, secondary antibodies were added to the slices in PB containing 0.1% Triton X-100 for 2 hours at room temperature. For the analysis of Purkinje cell dendritic size, rabbit anti-calbindin D-28K (Swant, Marly, Switzerland, 1 : 1000) was used as a primary antibody and goat anti-rabbit Alexa 568 (Molecular Probes, Invitrogen, 1 : 500) was used as a secondary antibody. To validate the expression of PMCA2 in Purkinje cell dendrites and dendritic spines, rabbit anti-calcium pump PMCA2 ATPase antibody (Abcam, United Kingdom, 1 : 500) was used as a primary antibody and goat anti-rabbit Alexa 488 (Molecular Probes, Invitrogen, 1 : 500) was used as a secondary antibody. For double-staining with PMCA2, a mouse monoclonal anti-calbindin D-28K antibody (Swant, Marly, Switzerland) was used as primary antibody and goat anti-mouse Alexa 568 as a secondary antibody. Stained slices were mounted on glass slides with coverslip using Mowiol. The microscopic observations were made on an Olympus AX-70 microscope equipped with a Spot Insight digital camera. Images were processed for optimization of brightness and contrast with Adobe Photoshop software.

### 2.3. Quantitative Analysis of Cultured Purkinje Cell Dendrites

The quantification of Purkinje cell dendritic tree size was done as previously described [5, 15]. Purkinje cells which had a dendritic tree not overlapping with neighboring cells were selected for analysis. An image analysis program (Image Pro Plus) was used to trace the outline of the Purkinje cell dendritic tree yielding the area covered by the dendritic tree. Purkinje cells were acquired from three independent experiments with an average number of 20 cells per experiment and per growth condition. For detailed information on the number of cells measured for each diagram see Table  1 in Supplementary Material available online at http://dx.doi.org/10.1155/2013/321685. 

For counting the number of branch points, one set of experimental data was used with approximately 20 cells per condition. The number of branch points was counted manually due to the highly branched and fine morphology of the Purkinje cell dendrites. A single Purkinje cell photograph taken with the 20x lens was zoomed and every branchpoint was counted and marked with a white dot using Adobe Photoshop [17].

The data were analyzed using GraphPad Prism software. The mean value of the dendritic tree area or the branchpoint number of untreated control cells were set to 100%, and the results were expressed as percentage of controls. Error bars represent the standard error of the mean (SEM). The statistical significance of differences in parameters was assessed by nonparametric analysis of variance (Kruskal-Wallis test) followed by Dunn's post test. For comparisons of single data columns, Mann-Whitney's nonparametric test was used. Confidence intervals were 95%; statistical significance was assumed with *P* < 0.05.

## 3. Results

### 3.1. The Plasma Membrane Ca^2+^-ATPase (PMCA2) Is Strongly Expressed in Purkinje Cell Dendrites in Cerebellar Slice Cultures

T- and P/Q-type Ca^2+^ channels are abundantly expressed in Purkinje cell dendrites [18, 19] and are one of the major sources of Ca^2+^ influx into Purkinje cells [18, 20–22]. Furthermore, Ca^2+^ influx through these channels has been shown to be potentiated by mGluR1 activation [19, 23, 24]. The plasma membrane Ca^2+^-ATPase (PMCA2) is abundantly and highly expressed in the Purkinje cell dendrites and dendritic spines [10, 11] and plays a crucial role in calcium dynamics and synaptic communication at cerebellar synapses [7].

**Figure 1:**
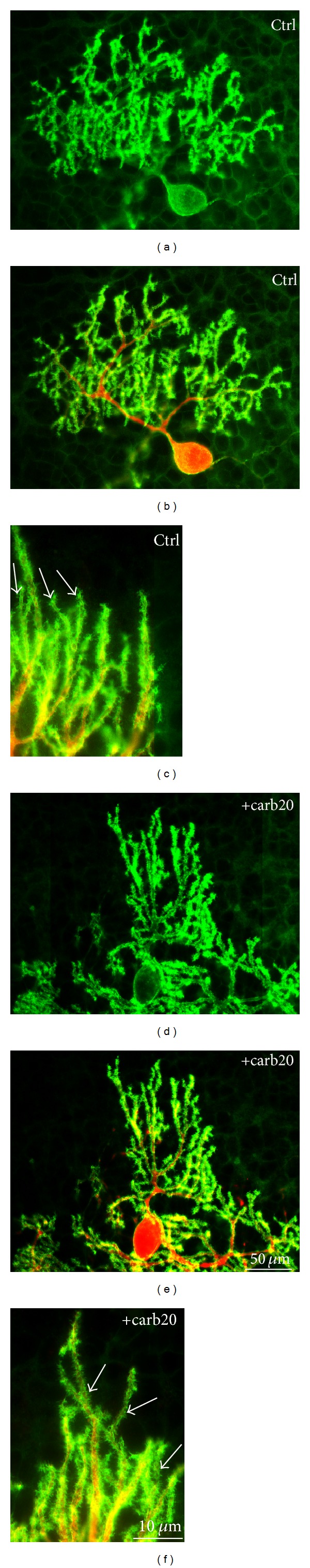
Staining of anti-calcium pump PMCA2 ATPase and calbindin D-28K in Purkinje cells in organotypic slice cultures: (a–c) untreated control cultures, (d–f) carboxyeosin-treated cultures. (a) Untreated control showing abundant expression of PMCA2 in dendrites and dendritic spines (green). (b) Merged red Purkinje cell soma with higher expression of PMCA2 in dendrites. (c) Abundant expression of PMCA2 in dendritic plasma membrane and dendritic spines. (d) Carboxyeosin-treated Purkinje cell showing abundant expression of PMCA2 in dendrites and dendritic spines (green). (e) Red Purkinje cell soma with higher expression of PMCA2 in dendrites and dendritic spines. (f) Abundant expression of PMCA2 in dendritic plasma membrane and dendritic spines. Scale bar 50 *μ*m (a-b, d-e) and 10 *μ*m (c–f).

In order to verify the expression of PMCA2 independently in Purkinje cell dendrites and dendritic spines in cerebellar slice cultures, we performed immunohistochemistry with anti-PMCA2 antibody on cerebellar slices after 10 DIV. We found that PMCA2 immunoreactivity (IR) was strongly present on the dendritic tree of the Purkinje cells, while there was only little expression on the axon, the cell soma, and the stem dendrites (Figure 1(a)). This became further evident in double-stainings with anti-calbindin which stained all parts of the Purkinje cells with a similar intensity. Therefore, the cell soma and proximal dendrites appear red in the double staining, whereas the distal dendritic tree appears yellow to green due to the strong presence of PMCA2 IR (Figure 1(b)). When the distal dendrites were viewed in high magnification, it could be seen that PMCA2 was strongly expressed at the dendritic plasma membrane and in dendritic spines of Purkinje cells (arrows in Figure 1(c)). When cerebellar slice cultures were treated chronically with the PMCA2 inhibitor carboxyeosin for 7 days, there was no major change in the expression pattern of PMCA2 in the Purkinje cell dendritic tree. PMCA2 staining remained strong and was concentrated in the plasma membrane of the peripheral part of the Purkinje cell dendritic tree (Figures 1(d)–1(f)) indicating that chronic inhibition of PMCA function did not affect the expression or cellular distribution of PMCA2 in a major way.

### 3.2. Chronic Inhibition of PMCA2 by Carboxyeosin Induced a Moderate Reduction of Purkinje Cell Dendritic Tree Size

**Figure 2:**
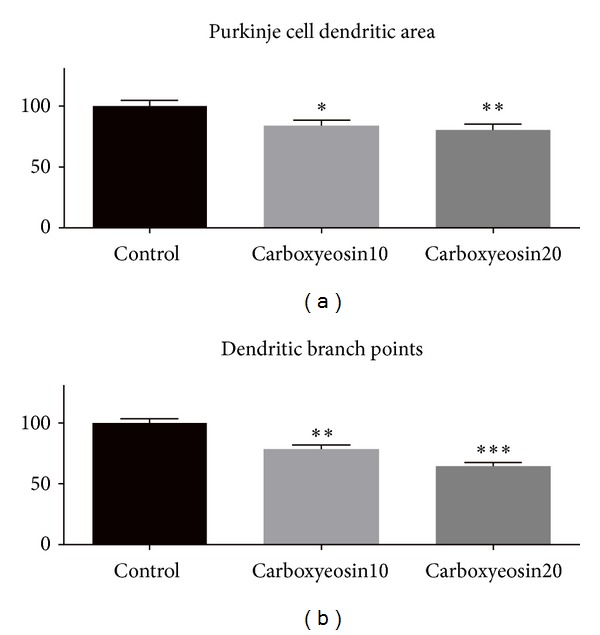
(a) Size of Purkinje cell dendritic arbors in control and carboxyeosin-treated cultures: The mean size of control Purkinje cells was considered as 100%. Purkinje cell dendritic trees from carboxyeosin-treated cultures were significantly smaller compared to control cultures with *P* < 0.05 (∗) for 10 *μ*M carboxyeosin and *P* < 0.01 (∗∗) for treatment with 20 *μ*M carboxyeosin. Error bars represent the SEM. (b) Number of branch points with carboxyeosin: The mean number of branch points of control Purkinje cells was considered as 100%. Carboxyeosin treatment alone showed a reduction of the number of branch points to 73% with 10 *μ*M and 62% with 20 *μ*M compared to control values. These differences were significant with *P* < 0.01 (∗∗) for 10 *μ*M carboxyeosin and with *P* < 0.001 (∗∗∗) for 20 *μ*M carboxyeosin. Error bars represent the SEM.

In studies with PMCA2 knockout mice, it was shown that Purkinje cells in these mice have a reduced size with a stunted dendritic tree [25] and have an increased resting calcium level [26]. We tested whether the chronic functional inhibition of PMCA2 in cerebellar slice cultures would also affect the development of the Purkinje cell dendritic tree. After 7 days of carboxyeosin treatment, we found a moderate but significant reduction of the Purkinje cell dendritic tree size by about 20% both for treatment with 10 *μ*M and 20 *μ*M carboxyeosin (Figure 2(a), supplemental Table 1) and a reduction of the number of branch points to 73% with 10 *μ*M and 62% with 20 *μ*M carboxyeosin (Figure 2, supplemental Table 2). This finding is in line with the observations in PMCA2 knockout mice [25] and shows that chronic carboxyeosin treatment mimics a loss of function of PMCA2 and that dendritic growth under these conditions is reduced. 

### 3.3. Chronic Inhibition of PMCA2 Protects Purkinje Cells from Dendritic Reduction Induced by mGluR1 Stimulation

**Figure 3:**
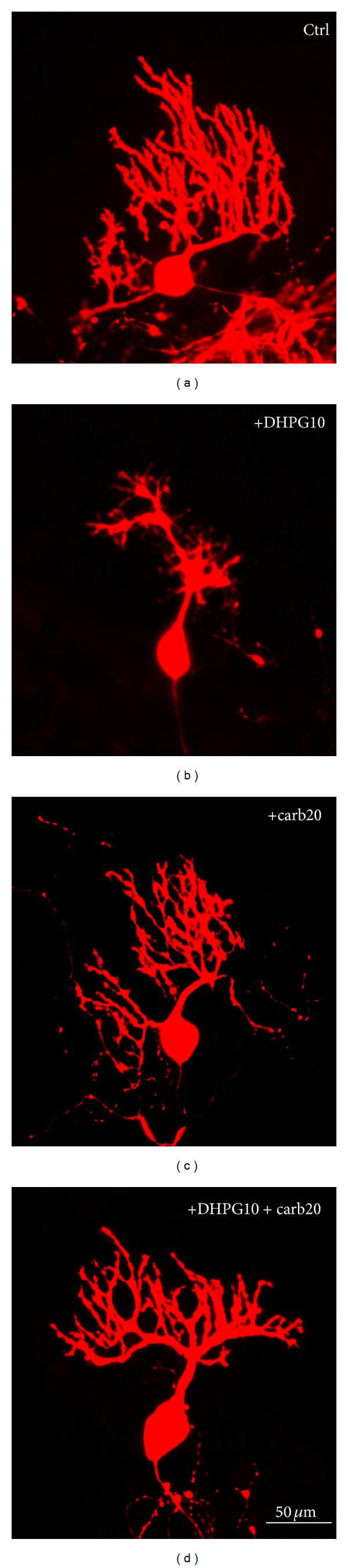
(a–d) Anti-calbindin-stained Purkinje cells from different pharmacological treatment. (a) Purkinje cells from untreated control cultures have a well-defined and profuse dendritic arbor. (b) Purkinje cells from slice culture treated with 10 *μ*M DHPG have a dendritic arbor greatly reduced in size with an absence of the distal dendritic branches. (c) Purkinje cells from carboxyeosin-treated cultures have a dendritic arbor smaller compared to untreated control but larger compared to DHPG treated cultures. (d) Cotreatment with 20 *μ*M carboxyeosin provides a partial rescue of the distal dendritic branches of the Purkinje cells. The size of the dendritic arbor is larger compared to DHPG treatment alone. Scale bar 50 *μ*m.

**Figure 4:**
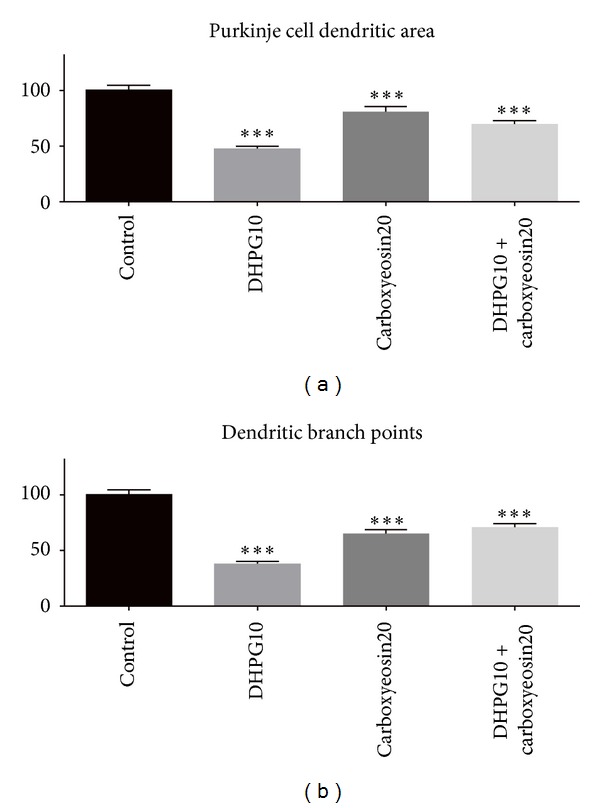
(a) Size of Purkinje cell dendritic arbors with different pharmacological treatments: The mean size of control Purkinje cells was considered as 100%. Dendritic tree size was reduced to 48% of control with DHPG treatment. Cotreatment with carboxyeosin resulted in a rescue of the dendritic area to 70% of the control value, similar to the size after treatment with carboxyeosin alone. These differences were significant with *P* < 0.001 (∗∗∗). Error bars represent the SEM. (b) Number of branch points with the pharmacological treatments: The mean number of branch points of control Purkinje cells was considered as 100%. The branchpoint number is reduced to 37% in DHPG treated cultures compared to control. Cotreatment with carboxyeosin showed a rescue of the branch points to 70% of the control value. These differences were significant with *P* < 0.001 (∗∗∗). Error bars represent the SEM.

As shown earlier [5, 6], chronic treatment of cerebellar slice cultures with the mGluR1 agonist DHPG induces a strong reduction of the Purkinje cell dendritic tree. Because PMCA2 is an important regulator of the calcium homeostasis in Purkinje cell dendrites, we studied whether the mGluR1 effect might be mediated by PMCA2. We pretreated cerebellar slice cultures with the PMCA2 inhibitor carboxyeosin for 1 day followed by coapplication of DHPG and carboxyeosin for 7 days. We found that pretreatment with carboxyeosin had a strong rescuing effect for the Purkinje cell dendritic tree after mGluR1 activation by DHPG treatment (Figure 3(d)). The dendritic tree of Purkinje cells from cotreated slice cultures appeared similar to that after carboxyeosin treatment alone (Figures 3(c) and 4(a)) but was much larger than after DHPG treatment alone (Figures 3(b) and 4(a)). Qualitatively, there was a reappearance of the peripheral Purkinje cell dendritic branches after cotreatment, and the overall shape of the dendritic tree resembled that of cells from carboxyeosin treatment alone (Figure 3(c)).

The quantitative data indicate that mGluR1 stimulation with DHPG gives rise to a reduction of the size of dendritic arbors to 48% of the size in untreated control cultures. In contrast, cotreatment with carboxyeosin resulted in a rescue of the dendritic area to 70% of that in untreated control cultures; that is, dendritic tree size was increased by 45% compared to DHPG treatment alone (Figure 4(a), supplemental Table 1). Furthermore, the branch-point number is reduced to 37% of the control value in DHPG-treated cultures. Cotreatment with carboxyeosin yielded a rescue of the branch points to 70% of the control value (Figure 4(b), supplemental Table 2). In fact, the size of Purkinje cell dendritic tree and the number of branch points after co-treatment with carboxyeosin and the mGluR1 agonist DHPG were similar to the values after treatment with carboxyeosin alone indicating that the mGluR1 activation resulted in no additional inhibition of dendritic growth. These differences were significant with *P* < 0.001 confirming the rescuing effect of carboxyeosin treatment for mGluR1-mediated dendritic reduction.

## 4. Discussion

The major finding of this study is that PMCA2 inhibition with carboxyeosin by itself reduced the size of the Purkinje cell dendritic tree, but at the same time it had a strong rescuing effect for the dendritic tree from chronic mGluR1 stimulation through DHPG. The finding that PMCA2 inhibition reduces Purkinje cell dendritic tree size confirms earlier observations with PMCA2 knockout mice [25]. The somewhat unexpected finding that PMCA2 inhibition protects Purkinje cells from mGluR1-mediated dendritic reduction highlights the importance of the calcium equilibrium for the control of Purkinje cell dendritic tree size during dendritic development.

Our previous work has shown that chronic mGluR1 stimulation during Purkinje cell dendritic development in cerebellar slice cultures induces a marked reduction of the Purkinje cell dendritic tree size [5] and Ca^2+^ influx through voltage-gated P/Q-type and T-type channels are crucial mediators of the DHPG-induced dendritic tree reduction [6]. In agreement with this concept, we had shown that inhibition of P/Q- and T-type Ca^2+^channels had a rescuing effect for the Purkinje cell dendrites after DHPG-treatment. Inhibiting PMCA2 by carboxyeosin will inhibit a major Ca^2+^-extrusion mechanism and would thus result in increased Ca^2+^ levels. This view is supported by the finding of increased resting levels of intracellular Ca^2+^ in Purkinje cells [26] and a reduced size of the Purkinje cell dendritic tree in the PMCA2 knockout mouse [25]. Our finding of a moderate reduction of Purkinje cell dendritic tree size after carboxyeosin treatment alone is in agreement with the previous observations and confirms the importance of PMCA2 for maintaining the Ca^2+^ equilibrium in Purkinje cell dendrites. The strong presence of PMCA2 at the plasma membrane of the distal dendritic tree and in dendritic spines of Purkinje cells underlines its important role for calcium homeostasis in the Purkinje cell dendrites. It is very likely that chronically elevated resting calcium levels induced by PMCA2 inhibition through carboxyeosin treatment had an inhibitory effect on dendritic growth. Because the size of the dendritic tree was only moderately reduced, it is likely to assume that the Purkinje cells activated compensatory mechanisms which could, to a large degree, counteract the reduction of Ca^2+^ extrusion induced by PMCA2 inhibition.

We had previously suggested that an increased Ca^2+^ influx and a rise of the intracellular Ca^2+^ concentration are the effector pathways for Purkinje cell dendritic reduction after mGluR1 stimulation by DHPG treatment. If this assumption was correct, then the protective effect of cotreatment of DHPG with carboxyeosin is somewhat surprising. Normally, a block of a major Ca^2+^ extrusion mechanism would be expected to result in an increased intracellular Ca^2+^ concentration and should lead to a potentiation of the DHPG effect. A possible explanation for our contradictory finding of a protective effect probably comes from the chronic nature of the drug treatments in this study, leading to the activation of compensatory mechanisms within the Purkinje cell dendrites.

In the wriggle mutant mouse, there is a point mutation in the PMCA2 gene resulting in a loss of function phenotype similar to the PMCA2 knockout mouse [13]. When depolarization-induced Ca^2+^ influx into Purkinje cells in these mice was studied, it was found to be greatly reduced quite in contrast to the expectation of an increased Ca^2+^ rise due to the reduced extrusion. The most likely explanation for this finding is a compensatory inactivation and downregulation of voltage-gated Ca^2+^ channels with a strong reduction of depolarization-induced Ca^2+^ influx as actually found in these mice [13]. In accordance with this explanation, it was shown that there was a dramatic reduction of both the frequency and amplitude of complex spikes and depolarization-induced Ca^2+^ influx in Purkinje cells from PMCA2 knockout mice [26]. It can be expected that a similar functional inactivation of voltage-gated Ca^2+^ channels did occur with the chronic carboxyeosin treatment. Thus, carboxyeosin treatment can be seen as an effective alternative way to inhibit voltage-gated Ca^2+^channels and reduce Ca^2+^ influx in Purkinje cells mediated by mGluR1 activation. This interpretation is supported by experiments in which we have combined carboxyeosin treatment with an additional block of P/Q-type and T-type Ca^2+^channels. As shown in supplemental Figure 1, the Ca^2+^channel block did provide a rescue from DHPG-mediated dendritic reduction as shown earlier [6]. In combination with carboxyeosin treatment, the additional Ca^2+^channel block affected neither the morphology nor the size of the Purkinje cell dendritic tree compared to carboxyeosin treatment alone (supplemental Figure 1). This outcome is expected if PMCA2 inhibition by itself does produce Ca^2+^channel inhibition confirming our interpretation that carboxyeosin treatment results in a functional inhibition of voltage-gated Ca^2+^channels in Purkinje cells.

## 5. Conclusions

We showed that chronic inhibition of PMCA2 results in a moderate reduction of Purkinje cell dendritic tree size but that in combination with DHPG treatment it confers a rescuing effect from chronic mGluR1 stimulation. This rescuing effect may appear surprising at the first glance, but it can be explained by an inhibition of voltage-gated Ca^2+^ channels with chronic PMCA2 inactivation and confirms our previous findings that a rescue of the Purkinje cell dendritic tree from mGluR1-induced reduction can be achieved by the inhibition of voltage-gated Ca^2+^ channels [6]. It further shows that PMCA2 is crucially involved in the maintenance and control of the calcium equilibrium in developing Purkinje cell dendrites and that this equilibrium is critical for the control of dendritic growth and expansion.

## Supplementary Material

The supplemental Figure 1 provides data of additional experiments suggesting that the effect of carboxyeosin is due to an inactivation of voltage-gated Ca^2+^channels. The supplemental tables contains the concrete figures of the measurements of dendritic area (supplemental table 1) and branchpoint numbers (supplemental table 2) shown in Figures. 2 and 4 of the manuscript.

## List of Abbreviations

Ca^2+^: Calcium
CB6: B6CF1
DHPG: Dihydroxyphenylglycine
Carboxyeosin: CEDA-SE 5(6)-Carboxyeosin diacetate ester
mGluR1: Metabotropic glutamate receptor, type 1
PMCA2: Plasma membrane Ca^2+^ ATPase, isoform 2
IR: Immunoreactivity
DIV: Days in vitro.
