# Supplementary material for: The Plasma Membrane Ca2+-ATPase2 (PMCA2) Is Involved in the Regulation of Purkinje Cell Dendritic Growth in Cerebellar Organotypic Slice Cultures
Source: Neural Plast. 2013 Sep 29;2013:321685. doi: 10.1155/2013/321685 (PMC3830849; doi:10.1155/2013/321685)
Supplement: Supplementary file 1 — The supplemental Figure 1 provides data of additional experiments suggesting that the effect of carboxyeosin is due to an inactivation of voltage-gated Ca2+channels. The supplemental tables contains the concrete figures of the measurements of dendritic area (supplemental table 1) and branchpoint numbers (supplemental table 2) shown in Figures. 2 and 4 of the manuscript. [file 321685.f1.zip › 321685.f1/321685.pdf]

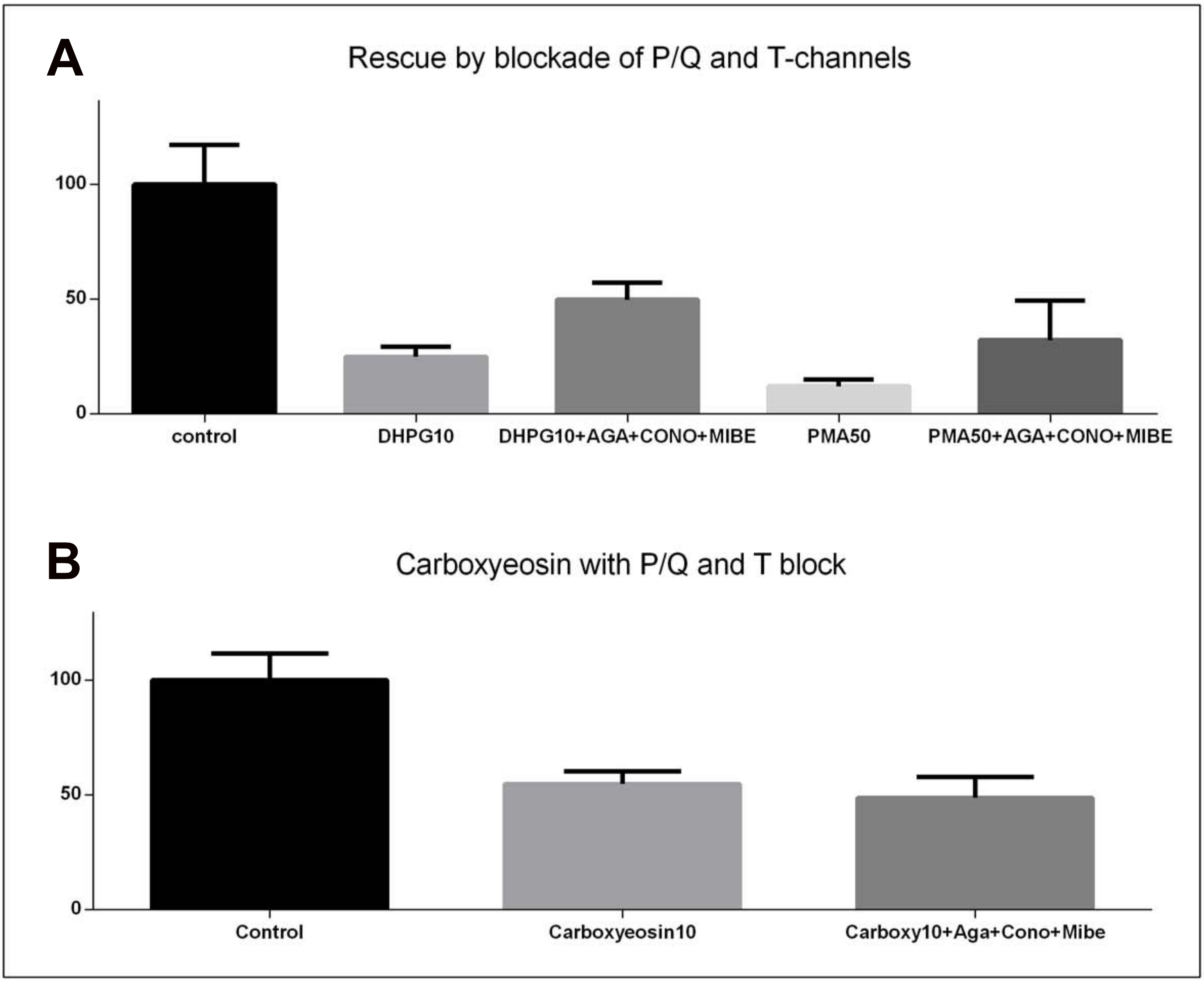
A
Rescue by blockade of P/Q and T-channels
100
50
0
control
DHPG10
DHPG10+AGA+CONO+MIBE
PMA50
PMA50+AGA+CONO+MIBE
B
Carboxyeosin with P/Q and T block
100
50
0
Control
Carboxyeosin10
Carboxy10+Aga+Cono+Mibe

Supplemental Fig. 1:

A) Size of the Purkinje cell dendritic arbors with blockade of P/Q-type and T-type Ca2+ channels and mGluR1/PKC stimulation: Blockade of P/Q-type and T-type Ca2+ channels with mGluR1 activation does provide a rescue from DHPG mediated dendritic reduction. Similarly, Ca2+ channels blockade shows similar dendritic rescue when PKC is activated by PMA. The mean size of control Purkinje cells was considered as 100%. Error bars represent the SEM. One set of experimental data with n= 15.

B) Size of the Purkinje cell dendritic arbors with blockade of P/Q-type and T-type Ca2+ channels and PMCA2 inhibition: The Purkinje cell dendritic arbors in with blockade of P/Q-type and T-type Ca2+ channels in addition to PMCA2 does not have an additive rescuing effect when PMCA2 was inhibited by carboxyeosin. The mean size of control Purkinje cells was considered as 100%. Error bars represent the SEM. One set of experimental data with n= 25.
