# Supplementary material for: The Plasma Membrane Ca2+-ATPase2 (PMCA2) Is Involved in the Regulation of Purkinje Cell Dendritic Growth in Cerebellar Organotypic Slice Cultures
Source: Neural Plast. 2013 Sep 29;2013:321685. doi: 10.1155/2013/321685 (PMC3830849; doi:10.1155/2013/321685)
Supplement: Supplementary file 1 — The supplemental Figure 1 provides data of additional experiments suggesting that the effect of carboxyeosin is due to an inactivation of voltage-gated Ca2+channels. The supplemental tables contains the concrete figures of the measurements of dendritic area (supplemental table 1) and branchpoint numbers (supplemental table 2) shown in Figures. 2 and 4 of the manuscript. [file 321685.f1.zip › 321685.f1/mat.321685.v2.pdf]

Table-1:

Statistics\conditions	control	Carboxyeosin10	carboxyeosin20	DHPG10	DHPG10 + Carboxyeosin20
Number of values	58	68	52	91	65
Mean dendritic area	3820	3201	3082	1816	2656
Std. Deviation	1355	1394	1309	658	965
Std. Error of Mean	177	169	181	69	119

Supplemental Table 1:

Measured values for the mean dendritic tree size of Purkinje cells in the different treatment conditions given in square micrometers. The "number of values" indicates the number measured Purkinje cell dendritic trees per treatment condition, the values for standard deviation and standard error of the mean indicate the variance of the data sets.

Table-2:

Statistics\conditions	control	Carboxyeosin10	carboxyeosin20	DHPG10	DHPG10 + Carboxyeosin20
Number of values	22	22	22	22	22
Mean branchpoints	30	23	19	11	21
Std. Deviation	5	5	4	3	4
Std. Error of Mean	1	1	1	1	1

Supplemental Table 2:

Measured values for the mean dendritic branch points of Purkinje cells from different treatment conditions. The "number of values" indicates the number of quantified Purkinje cells for their dendritic branch points per treatment condition, the values for standard deviation and standard error of the mean indicate the variance of the data sets.
